# *N*-(2,4,5-Trichloro­phen­yl)maleamic acid

**DOI:** 10.1107/S1600536809052520

**Published:** 2009-12-16

**Authors:** B. Thimme Gowda, Miroslav Tokarčík, Jozef Kožíšek, K. Shakuntala, Hartmut Fuess

**Affiliations:** a Department of Chemistry, Mangalore University, Mangalagangotri 574 199, Mangalore, India; b Faculty of Chemical and Food Technology, Slovak Technical University, Radlinského 9, SK-812 37 Bratislava, Slovak Republic; c Institute of Materials Science, Darmstadt University of Technology, Petersenstrasse 23, D-64287 Darmstadt, Germany

## Abstract

The title compound, C_10_H_6_Cl_3_NO_3_, crystallizes with two independent mol­ecules in the asymmetric unit. The mol­ecular structure is stabilized by a short intra­molecular O—H⋯O hydrogen bond within the maleamic unit. In the crystal, each mol­ecule self-associates *via* N—H⋯O hydrogen bonds into chains, each running along the *b* axis. Two short inter­molecular Cl⋯O contacts [3.1267 (15) and 3.0523 (12) Å] and C—H⋯O inter­actions inter­connect these chains into a three-dimensional network.

## Related literature

For studies on the effect of ring- and side-chain substitutions on the crystal structures of amides, see: Gowda *et al.* (2009 ▶, 2010 ▶); Lo & Ng (2009 ▶); Prasad *et al.* (2002 ▶); Shakuntala *et al.* (2009 ▶). For the concept of orthogonality of halogen and hydrogen bonds, see: Voth *et al.* (2009 ▶). For a review on short halogen–oxygen contacts, see: Fourmigué (2009 ▶); Kubicki (2004 ▶).
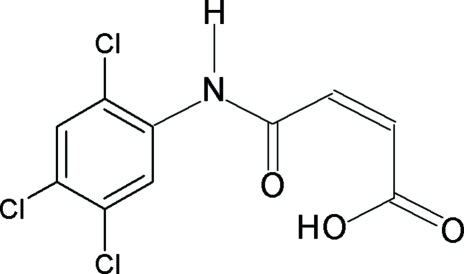


## Experimental

### 

#### Crystal data

C_10_H_6_Cl_3_NO_3_
*M* *_r_* = 294.51
Monoclinic, 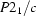

*a* = 10.8979 (2) Å
*b* = 11.0225 (2) Å
*c* = 19.4739 (3) Å
β = 95.4761 (9)°
*V* = 2328.57 (7) Å^3^
*Z* = 8
Mo *K*α radiation
μ = 0.78 mm^−1^
*T* = 295 K
0.34 × 0.25 × 0.22 mm

#### Data collection

Oxford Diffraction Xcalibur diffractometer with a Ruby Gemini detector
Absorption correction: analytical (*CrysAlis PRO*; Oxford Diffraction, 2009 ▶) *T*_min_ = 0.794, *T*_max_ = 0.852
49446 measured reflections
4424 independent reflections
3825 reflections with *I* > 2σ(*I*)
*R*_int_ = 0.022

#### Refinement

*R*[*F* ^2^ > 2σ(*F* ^2^)] = 0.028
*wR*(*F* ^2^) = 0.079
*S* = 1.06
4424 reflections
315 parameters
H atoms treated by a mixture of independent and constrained refinement
Δρ_max_ = 0.28 e Å^−3^
Δρ_min_ = −0.32 e Å^−3^

### 

Data collection: *CrysAlis PRO* (Oxford Diffraction, 2009 ▶); cell refinement: *CrysAlis PRO*; data reduction: *CrysAlis PRO*; program(s) used to solve structure: *SHELXS97* (Sheldrick, 2008 ▶); program(s) used to refine structure: *SHELXL97* (Sheldrick, 2008 ▶); molecular graphics: *ORTEP-3* (Farrugia, 1997 ▶) and *DIAMOND* (Brandenburg, 2002 ▶); software used to prepare material for publication: *SHELXL97*, *PLATON* (Spek, 2009 ▶) and *WinGX* (Farrugia, 1999 ▶).

## Supplementary Material

Crystal structure: contains datablocks I, global. DOI: 10.1107/S1600536809052520/tk2591sup1.cif

Structure factors: contains datablocks I. DOI: 10.1107/S1600536809052520/tk2591Isup2.hkl

Additional supplementary materials:  crystallographic information; 3D view; checkCIF report

## Figures and Tables

**Table 1:** Hydrogen-bond geometry (Å, °)

*D*—H⋯*A*	*D*—H	H⋯*A*	*D*⋯*A*	*D*—H⋯*A*
O2—H2*A*⋯O1	0.82	1.69	2.5080 (15)	175
O5—H5*A*⋯O4	0.82	1.72	2.5332 (17)	175
N1—H1*N*⋯O3^i^	0.77 (2)	2.30 (2)	3.0356 (18)	161.2 (19)
N2—H2*N*⋯O6^ii^	0.81 (2)	2.481 (19)	3.0775 (19)	131.3 (16)
N1—H1*N*⋯Cl1	0.77 (2)	2.56 (2)	2.9628 (14)	114.2 (17)
N2—H2*N*⋯Cl4	0.81 (2)	2.519 (18)	2.9476 (14)	114.3 (15)
C2—H2⋯O3^i^	0.93	2.29	3.123 (2)	149
C12—H12⋯O2	0.93	2.47	3.330 (2)	154

Symmetry codes: (i) 
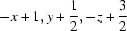
; (ii) 
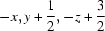
.

**Table 2:** Halogen-bond geometry (Å, °)

C—Cl⋯O	Cl⋯O	C—Cl⋯O
C9—Cl3⋯O6^iii^	3.1267 (15)	158.99 (6)
C16—Cl4⋯O1	3.0523 (12)	159.62 (7)

Symmetry code: (iii) 
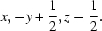

**Fig. 1.**
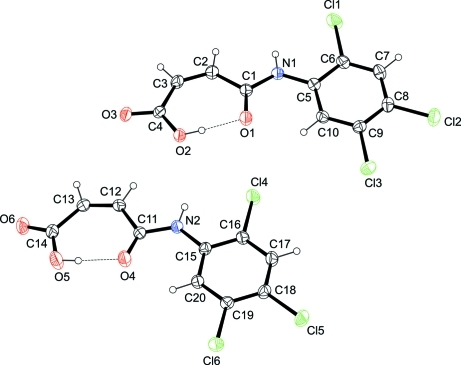
Molecular structure of the two independent molecules comprising the asymmetric unit in (I) showing the atom labelling scheme. Displacement ellipsoids are drawn at the 30% probability level. H atoms are represented as small spheres of arbitrary radii.

**Fig. 2.**
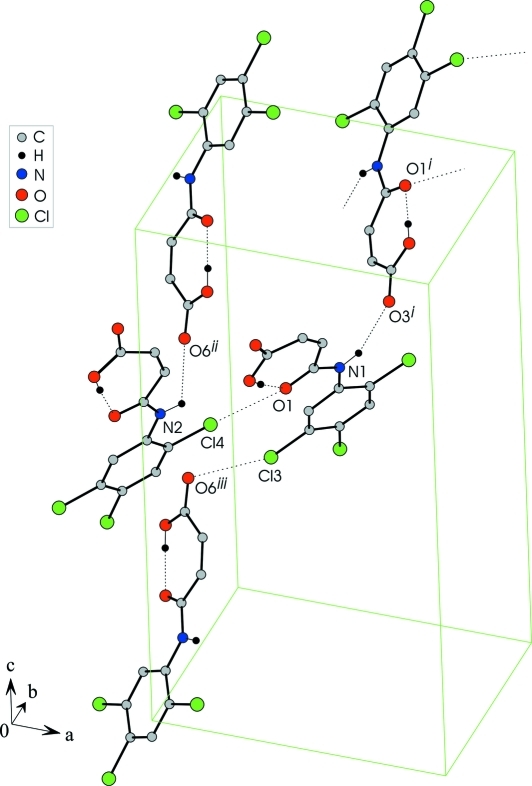
Part of crystal structure of (I) showing the main intermolecular interactions: N–H···O hydrogen bonds and short Cl···O contacts (all represented by dashed lines). Symmetry codes: (i) -x + 1, y + 1/2, -z + 3/2; (ii) -x, y + 1/2, -z + 3/2; (iii) x, -y + 1/2, z - 1/2.

## supplementary crystallographic information

### Comment

In the present work, as a part of studying the effect of ring- and side-chain substitutions on the crystal structures of biologically significant amides (Gowda *et al.*, 2009, 2010; Shakuntala *et al.*, 2009; Prasad *et al.*, 2002), the crystal structure of *N*-(2,4,5-trichlorophenyl)-maleamic acid (I) has been determined. The asymmetric unit of (I) contains two independent molecules (Fig. 1).

The conformations of the N—H and C=O bonds in the amide segment of the structure are *anti* to each other, and the amide-O atom and the carbonyl-O atom of the acid segment are also *anti* to each other. Further, the amide-O atom is *anti* to the H atom attached to the adjacent C atom, while the carbonyl-O atom is *syn* to the H atom attached to its adjacent C atom (Fig.1). In the structure, a rare *anti* conformation of the C=O and O—H bonds of the acid group has been observed, similar to that observed in *N*-phenylmaleamic acid (Lo & Ng, 2009), *N*-(2,5-dichlorophenyl)maleamic acid (Shakuntala *et al.*, 2009), *N*-(3,5-dichlorophenyl)maleamic acid (Gowda *et al.*, 2010) and *N*-(2,4,6-dimethylphenyl)maleamic acid (Gowda *et al.*, 2009). Further, the conformation of the N—H bond is *syn* to the 2-Cl in the phenyl ring, while it is *anti* to the 5-Cl in the ring. Each maleamic unit includes a short intramolecular hydrogen O—H···O bond (Table 1). The bond lengths of C2–C3 and C12–C13 (i.e. 1.329 (2) and 1.328 (2) Å) clearly indicate double bond character.

In both the molecules, the dihedral angle between the amido group –NHCO– and the tri-substituted phenyl ring is 6.1 (3)°. The two N—H···O hydrogen bonds have quite different geometry, as seen in the N—H···O angles (Table 1). Remarkably small N2—H2N···O6^ii^ angle of 131.3 (16)° can be attributed to the competing effect of the neighbouring C9—Cl3···O6^iii^ halogen bond. The angle Cl3···O6^iii^···H2N =78.24 (6)° is close to 90° and can be interpreted within the concept of orthogonality of halogen and hydrogen bonds (Voth *et al.*, 2009). The N1—H1N···O3^i^ hydrogen bond with the angle of 161.2 (19)° is much less influenced by the near C6—Cl1···O3^i^ halogen bond, which is weaker as indicated by the Cl1···O3^i^ contact of 3.2372 (13) A. [Symmetry codes: (i) -*x* + 1, *y* + 1/2, -*z* + 3/2; (ii) -*x*, *y* + 1/2, -*z* + 3/2; (iii) *x*, -*y* + 1/2, *z* - 1/2.]

In the crystal of (I), the intermolecular N–H···O hydrogen bonds link the molecules, which self-associate, into infinite chains running along the *b* axis. These chains are connected through relatively short Cl···O contacts: Cl4···O1 =3.0523 (12), Cl3···O6(iii) =3.1267 (15) Å. [Symmetry codes: (iii) *x*, -*y* + 1/2, *z* - 1/2].(iii) *x*, -*y* + 1/2, *z* - 1/2] as well as C–H···O contacts to consolidate the crystal packing. Fig. 2 outlines part of the crystal structure of (I) with the N–H···O hydrogen bonding and Cl···O contacts highlighted. The data for the C–Cl···O halogen bonds are in agreement with others (Kubicki, 2004; Fourmigué, 2009).

### Experimental

The solution of maleic anhydride (0.025 mol) in toluene (25 ml) was treated drop-wise with the solution of 2,4,5-trichloroaniline (0.025 mol) also in toluene (20 ml) with constant stirring. The resulting mixture was warmed with stirring for over 30 min and set aside for an additional 30 min at room temperature for completion of the reaction. The mixture was then treated with dilute hydrochloric acid to remove the unreacted 2,4,5-trichloroaniline. The resultant solid *N*-(2,4,5-trichlorophenyl)maleamic acid was filtered under suction and washed thoroughly with water to remove the unreacted maleic anhydride and maleic acid. It was recrystallized to constant melting point from ethanol. The purity of the compound was checked by elemental analysis and characterized by its infrared spectra. Colourless single crystals used in X-ray diffraction studies were grown in an ethanol solution by slow evaporation at room temperature.

### Refinement

H atoms were visible in difference maps. In the final cycles of refinement, the amido-H atoms were refined freely, while the C,*O*-bound H atoms were placed in calculated positions and refined using the riding model with C–H = 0.93Å and O–H = 0.82 Å. The *U*_iso_(H) values were set at 1.2*U*_eq_(C,*O*).

### Crystal data

**Table:**

C_10_H_6_Cl_3_NO_3_	*F*(000) = 1184
*M**_r_* = 294.51	*D*_x_ = 1.68 Mg m^−^^3^
Monoclinic, *P*2_1_/*c*	Mo *K*α radiation, λ = 0.71073 Å
Hall symbol: -P 2ybc	Cell parameters from 30027 reflections
*a* = 10.8979 (2) Å	θ = 1.8–29.5°
*b* = 11.0225 (2) Å	µ = 0.78 mm^−^^1^
*c* = 19.4739 (3) Å	*T* = 295 K
β = 95.4761 (9)°	Block, colourless
*V* = 2328.57 (7) Å^3^	0.34 × 0.25 × 0.22 mm
*Z* = 8	

### Data collection

**Table:**

Oxford Diffraction Xcalibur diffractometer with a Ruby Gemini detector	4424 independent reflections
graphite	3825 reflections with *I* > 2σ(*I*)
Detector resolution: 10.434 pixels mm^-1^	*R*_int_ = 0.022
ω scans	θ_max_ = 25.7°, θ_min_ = 1.9°
Absorption correction: analytical (*CrysAlis PRO*; Oxford Diffraction, 2009)	*h* = −13→13
*T*_min_ = 0.794, *T*_max_ = 0.852	*k* = −13→13
49446 measured reflections	*l* = −23→23

### Refinement

**Table:**

Refinement on *F*^2^	Primary atom site location: structure-invariant direct methods
Least-squares matrix: full	Secondary atom site location: difference Fourier map
*R*[*F*^2^ > 2σ(*F*^2^)] = 0.028	Hydrogen site location: inferred from neighbouring sites
*wR*(*F*^2^) = 0.079	H atoms treated by a mixture of independent and constrained refinement
*S* = 1.06	*w* = 1/[σ^2^(*F*_o_^2^) + (0.0464*P*)^2^ + 0.5233*P*] where *P* = (*F*_o_^2^ + 2*F*_c_^2^)/3
4424 reflections	(Δ/σ)_max_ = 0.001
315 parameters	Δρ_max_ = 0.28 e Å^−^^3^
0 restraints	Δρ_min_ = −0.32 e Å^−^^3^

### Special details

**Table:**

Geometry. All e.s.d.'s (except the e.s.d. in the dihedral angle between two l.s. planes) are estimated using the full covariance matrix. The cell e.s.d.'s are taken into account individually in the estimation of e.s.d.'s in distances, angles and torsion angles; correlations between e.s.d.'s in cell parameters are only used when they are defined by crystal symmetry. An approximate (isotropic) treatment of cell e.s.d.'s is used for estimating e.s.d.'s involving l.s. planes.
Refinement. Refinement of *F*^2^ against ALL reflections. The weighted *R*-factor *wR* and goodness of fit *S* are based on *F*^2^, conventional *R*-factors *R* are based on *F*, with *F* set to zero for negative *F*^2^. The threshold expression of *F*^2^ > σ(*F*^2^) is used only for calculating *R*-factors(gt) *etc*. and is not relevant to the choice of reflections for refinement. *R*-factors based on *F*^2^ are statistically about twice as large as those based on *F*, and *R*- factors based on ALL data will be even larger.

### Fractional atomic coordinates and isotropic or equivalent isotropic displacement parameters (Å^2^)

**Table:**

	*x*	*y*	*z*	*U*_iso_*/*U*_eq_	
C1	0.44390 (14)	0.49367 (14)	0.63260 (8)	0.0359 (3)	
C2	0.49027 (16)	0.44723 (15)	0.70185 (8)	0.0435 (4)	
H2	0.5531	0.4921	0.7254	0.052*	
C3	0.45354 (16)	0.34984 (16)	0.73449 (8)	0.0437 (4)	
H3	0.4945	0.3395	0.7782	0.052*	
C4	0.36034 (14)	0.25383 (14)	0.71553 (8)	0.0353 (3)	
C5	0.47664 (14)	0.65859 (13)	0.55063 (8)	0.0338 (3)	
C6	0.55197 (15)	0.75667 (14)	0.53696 (8)	0.0390 (4)	
C7	0.52840 (17)	0.82580 (15)	0.47808 (9)	0.0447 (4)	
H7	0.5787	0.8917	0.4705	0.054*	
C8	0.43056 (16)	0.79770 (15)	0.43042 (8)	0.0418 (4)	
C9	0.35753 (15)	0.69853 (15)	0.44191 (8)	0.0385 (4)	
C10	0.37961 (15)	0.63019 (14)	0.50161 (8)	0.0374 (3)	
H10	0.3289	0.5645	0.5090	0.045*	
N1	0.50133 (13)	0.59440 (12)	0.61283 (7)	0.0364 (3)	
H1N	0.5504 (19)	0.6210 (18)	0.6398 (10)	0.047 (6)*	
O1	0.36099 (12)	0.44379 (11)	0.59559 (6)	0.0524 (3)	
O2	0.29633 (10)	0.25568 (10)	0.65501 (6)	0.0440 (3)	
H2A	0.3156	0.3154	0.6334	0.053*	
O3	0.34614 (11)	0.17281 (10)	0.75610 (6)	0.0439 (3)	
Cl1	0.67690 (5)	0.79432 (5)	0.59434 (2)	0.06029 (15)	
Cl2	0.40225 (5)	0.88690 (4)	0.35759 (3)	0.06031 (15)	
Cl3	0.23559 (4)	0.65766 (5)	0.38355 (2)	0.05600 (14)	
C11	−0.01578 (14)	0.08037 (14)	0.62405 (8)	0.0356 (3)	
C12	0.05760 (15)	0.08711 (15)	0.69170 (8)	0.0405 (4)	
H12	0.1200	0.1450	0.6953	0.049*	
C13	0.04666 (15)	0.02176 (16)	0.74809 (8)	0.0429 (4)	
H13	0.1058	0.0398	0.7843	0.051*	
C14	−0.04107 (16)	−0.07430 (17)	0.76485 (9)	0.0468 (4)	
C15	−0.04415 (14)	0.19115 (14)	0.51309 (7)	0.0348 (3)	
C16	−0.00319 (16)	0.29029 (15)	0.47665 (8)	0.0393 (4)	
C17	−0.05759 (17)	0.32085 (16)	0.41215 (8)	0.0450 (4)	
H17	−0.0298	0.3880	0.3892	0.054*	
C18	−0.15340 (16)	0.25164 (15)	0.38159 (8)	0.0410 (4)	
C19	−0.19375 (15)	0.15206 (15)	0.41656 (8)	0.0374 (3)	
C20	−0.14062 (15)	0.12225 (14)	0.48168 (8)	0.0380 (4)	
H20	−0.1696	0.0557	0.5046	0.046*	
N2	0.01228 (14)	0.16733 (13)	0.57959 (7)	0.0393 (3)	
H2N	0.0613 (18)	0.2172 (17)	0.5962 (9)	0.044 (5)*	
O4	−0.09307 (13)	0.00116 (12)	0.60847 (6)	0.0572 (4)	
O5	−0.12126 (16)	−0.11735 (15)	0.71730 (7)	0.0818 (5)	
H5A	−0.1146	−0.0818	0.6808	0.098*	
O6	−0.03687 (14)	−0.11161 (13)	0.82354 (7)	0.0629 (4)	
Cl4	0.11807 (5)	0.37788 (4)	0.51289 (2)	0.05662 (14)	
Cl5	−0.22020 (5)	0.29035 (5)	0.30038 (2)	0.06325 (15)	
Cl6	−0.31341 (4)	0.06298 (4)	0.38018 (2)	0.05475 (14)	

### Atomic displacement parameters (Å^2^)

**Table:**

	*U*^11^	*U*^22^	*U*^33^	*U*^12^	*U*^13^	*U*^23^
C1	0.0387 (8)	0.0346 (8)	0.0336 (8)	−0.0062 (6)	−0.0006 (6)	−0.0020 (6)
C2	0.0481 (10)	0.0433 (9)	0.0364 (8)	−0.0128 (7)	−0.0103 (7)	−0.0004 (7)
C3	0.0486 (10)	0.0459 (9)	0.0335 (8)	−0.0075 (8)	−0.0123 (7)	0.0040 (7)
C4	0.0341 (8)	0.0345 (8)	0.0363 (8)	0.0035 (6)	−0.0014 (6)	0.0018 (7)
C5	0.0366 (8)	0.0325 (8)	0.0326 (7)	−0.0023 (6)	0.0044 (6)	−0.0035 (6)
C6	0.0420 (9)	0.0372 (8)	0.0376 (8)	−0.0096 (7)	0.0035 (7)	−0.0066 (7)
C7	0.0546 (10)	0.0372 (9)	0.0435 (9)	−0.0113 (7)	0.0107 (8)	−0.0004 (7)
C8	0.0498 (10)	0.0393 (9)	0.0372 (8)	0.0034 (7)	0.0095 (7)	0.0047 (7)
C9	0.0371 (8)	0.0426 (9)	0.0354 (8)	0.0011 (7)	0.0007 (7)	−0.0016 (7)
C10	0.0367 (8)	0.0378 (8)	0.0373 (8)	−0.0074 (7)	0.0012 (7)	0.0012 (7)
N1	0.0387 (7)	0.0359 (7)	0.0331 (7)	−0.0101 (6)	−0.0049 (6)	−0.0018 (6)
O1	0.0594 (8)	0.0510 (7)	0.0427 (6)	−0.0255 (6)	−0.0165 (6)	0.0126 (5)
O2	0.0467 (7)	0.0429 (6)	0.0398 (6)	−0.0156 (5)	−0.0099 (5)	0.0095 (5)
O3	0.0487 (7)	0.0372 (6)	0.0441 (6)	−0.0003 (5)	−0.0035 (5)	0.0105 (5)
Cl1	0.0609 (3)	0.0657 (3)	0.0520 (3)	−0.0323 (2)	−0.0066 (2)	−0.0027 (2)
Cl2	0.0741 (3)	0.0548 (3)	0.0516 (3)	0.0010 (2)	0.0036 (2)	0.0191 (2)
Cl3	0.0502 (3)	0.0696 (3)	0.0451 (2)	−0.0060 (2)	−0.01197 (19)	0.0054 (2)
C11	0.0355 (8)	0.0389 (8)	0.0320 (8)	−0.0030 (7)	0.0002 (6)	0.0020 (6)
C12	0.0358 (8)	0.0466 (9)	0.0377 (8)	−0.0102 (7)	−0.0041 (7)	0.0043 (7)
C13	0.0402 (9)	0.0516 (10)	0.0347 (8)	−0.0073 (7)	−0.0081 (7)	0.0046 (7)
C14	0.0491 (10)	0.0495 (10)	0.0403 (9)	−0.0074 (8)	−0.0043 (8)	0.0098 (8)
C15	0.0392 (8)	0.0366 (8)	0.0278 (7)	−0.0015 (6)	−0.0002 (6)	−0.0009 (6)
C16	0.0424 (9)	0.0405 (9)	0.0341 (8)	−0.0096 (7)	−0.0008 (7)	−0.0007 (7)
C17	0.0541 (10)	0.0454 (9)	0.0346 (8)	−0.0102 (8)	−0.0006 (7)	0.0069 (7)
C18	0.0465 (9)	0.0473 (9)	0.0282 (7)	−0.0003 (7)	−0.0024 (7)	0.0022 (7)
C19	0.0386 (8)	0.0404 (9)	0.0325 (8)	−0.0033 (7)	−0.0006 (6)	−0.0039 (6)
C20	0.0419 (9)	0.0375 (8)	0.0339 (8)	−0.0056 (7)	0.0006 (7)	0.0010 (6)
N2	0.0435 (8)	0.0411 (8)	0.0316 (7)	−0.0117 (6)	−0.0057 (6)	0.0031 (6)
O4	0.0698 (8)	0.0597 (8)	0.0385 (6)	−0.0307 (7)	−0.0139 (6)	0.0111 (6)
O5	0.0967 (12)	0.0929 (12)	0.0499 (8)	−0.0596 (10)	−0.0235 (8)	0.0280 (8)
O6	0.0715 (9)	0.0722 (9)	0.0433 (7)	−0.0173 (7)	−0.0040 (6)	0.0210 (6)
Cl4	0.0632 (3)	0.0586 (3)	0.0450 (2)	−0.0288 (2)	−0.0107 (2)	0.0078 (2)
Cl5	0.0709 (3)	0.0774 (3)	0.0373 (2)	−0.0156 (3)	−0.0160 (2)	0.0162 (2)
Cl6	0.0557 (3)	0.0606 (3)	0.0443 (2)	−0.0203 (2)	−0.0140 (2)	0.0024 (2)

### Geometric parameters (Å, °)

**Table:**

C1—O1	1.2304 (18)	C11—O4	1.2309 (19)
C1—N1	1.349 (2)	C11—N2	1.346 (2)
C1—C2	1.485 (2)	C11—C12	1.476 (2)
C2—C3	1.329 (2)	C12—C13	1.328 (2)
C2—H2	0.9300	C12—H12	0.9300
C3—C4	1.489 (2)	C13—C14	1.484 (2)
C3—H3	0.9300	C13—H13	0.9300
C4—O3	1.2121 (18)	C14—O6	1.211 (2)
C4—O2	1.3111 (18)	C14—O5	1.301 (2)
C5—C10	1.391 (2)	C15—C20	1.390 (2)
C5—C6	1.398 (2)	C15—C16	1.399 (2)
C5—N1	1.406 (2)	C15—N2	1.404 (2)
C6—C7	1.380 (2)	C16—C17	1.379 (2)
C6—Cl1	1.7274 (16)	C16—Cl4	1.7315 (16)
C7—C8	1.380 (3)	C17—C18	1.381 (2)
C7—H7	0.9300	C17—H17	0.9300
C8—C9	1.383 (2)	C18—C19	1.385 (2)
C8—Cl2	1.7292 (16)	C18—Cl5	1.7301 (16)
C9—C10	1.387 (2)	C19—C20	1.382 (2)
C9—Cl3	1.7240 (16)	C19—Cl6	1.7292 (16)
C10—H10	0.9300	C20—H20	0.9300
N1—H1N	0.77 (2)	N2—H2N	0.81 (2)
O2—H2A	0.8200	O5—H5A	0.8200
			
O1—C1—N1	122.38 (14)	O4—C11—N2	122.58 (14)
O1—C1—C2	123.03 (14)	O4—C11—C12	123.73 (14)
N1—C1—C2	114.58 (13)	N2—C11—C12	113.65 (14)
C3—C2—C1	128.39 (15)	C13—C12—C11	128.82 (15)
C3—C2—H2	115.8	C13—C12—H12	115.6
C1—C2—H2	115.8	C11—C12—H12	115.6
C2—C3—C4	133.25 (15)	C12—C13—C14	132.80 (15)
C2—C3—H3	113.4	C12—C13—H13	113.6
C4—C3—H3	113.4	C14—C13—H13	113.6
O3—C4—O2	120.49 (14)	O6—C14—O5	120.67 (16)
O3—C4—C3	119.25 (14)	O6—C14—C13	118.86 (16)
O2—C4—C3	120.25 (13)	O5—C14—C13	120.47 (15)
C10—C5—C6	117.71 (14)	C20—C15—C16	117.90 (14)
C10—C5—N1	123.25 (14)	C20—C15—N2	123.35 (14)
C6—C5—N1	119.04 (14)	C16—C15—N2	118.74 (14)
C7—C6—C5	121.26 (15)	C17—C16—C15	121.51 (15)
C7—C6—Cl1	118.52 (13)	C17—C16—Cl4	118.70 (13)
C5—C6—Cl1	120.22 (12)	C15—C16—Cl4	119.79 (12)
C6—C7—C8	120.35 (15)	C16—C17—C18	119.95 (15)
C6—C7—H7	119.8	C16—C17—H17	120.0
C8—C7—H7	119.8	C18—C17—H17	120.0
C7—C8—C9	119.22 (15)	C17—C18—C19	119.22 (14)
C7—C8—Cl2	119.33 (13)	C17—C18—Cl5	119.50 (13)
C9—C8—Cl2	121.46 (13)	C19—C18—Cl5	121.28 (13)
C8—C9—C10	120.56 (15)	C20—C19—C18	120.96 (15)
C8—C9—Cl3	121.27 (13)	C20—C19—Cl6	118.32 (12)
C10—C9—Cl3	118.16 (13)	C18—C19—Cl6	120.72 (12)
C9—C10—C5	120.85 (14)	C19—C20—C15	120.44 (15)
C9—C10—H10	119.6	C19—C20—H20	119.8
C5—C10—H10	119.6	C15—C20—H20	119.8
C1—N1—C5	127.17 (14)	C11—N2—C15	128.30 (14)
C1—N1—H1N	115.5 (15)	C11—N2—H2N	114.0 (13)
C5—N1—H1N	117.3 (15)	C15—N2—H2N	117.0 (13)
C4—O2—H2A	109.5	C14—O5—H5A	109.5
			
O1—C1—C2—C3	0.1 (3)	O4—C11—C12—C13	−7.6 (3)
N1—C1—C2—C3	−178.97 (18)	N2—C11—C12—C13	174.36 (18)
C1—C2—C3—C4	1.6 (3)	C11—C12—C13—C14	−2.5 (3)
C2—C3—C4—O3	179.39 (19)	C12—C13—C14—O6	−173.1 (2)
C2—C3—C4—O2	0.8 (3)	C12—C13—C14—O5	6.7 (3)
C10—C5—C6—C7	2.3 (2)	C20—C15—C16—C17	1.2 (2)
N1—C5—C6—C7	−177.16 (15)	N2—C15—C16—C17	−177.78 (16)
C10—C5—C6—Cl1	−178.08 (12)	C20—C15—C16—Cl4	−179.11 (13)
N1—C5—C6—Cl1	2.4 (2)	N2—C15—C16—Cl4	1.9 (2)
C5—C6—C7—C8	−1.3 (3)	C15—C16—C17—C18	−1.2 (3)
Cl1—C6—C7—C8	179.06 (13)	Cl4—C16—C17—C18	179.12 (14)
C6—C7—C8—C9	−0.9 (3)	C16—C17—C18—C19	0.1 (3)
C6—C7—C8—Cl2	179.30 (13)	C16—C17—C18—Cl5	−179.56 (14)
C7—C8—C9—C10	2.0 (2)	C17—C18—C19—C20	0.8 (3)
Cl2—C8—C9—C10	−178.14 (13)	Cl5—C18—C19—C20	−179.46 (13)
C7—C8—C9—Cl3	−178.97 (13)	C17—C18—C19—Cl6	180.00 (14)
Cl2—C8—C9—Cl3	0.9 (2)	Cl5—C18—C19—Cl6	−0.3 (2)
C8—C9—C10—C5	−1.0 (2)	C18—C19—C20—C15	−0.8 (3)
Cl3—C9—C10—C5	179.95 (12)	Cl6—C19—C20—C15	−179.99 (12)
C6—C5—C10—C9	−1.1 (2)	C16—C15—C20—C19	−0.2 (2)
N1—C5—C10—C9	178.32 (15)	N2—C15—C20—C19	178.71 (15)
O1—C1—N1—C5	1.4 (3)	O4—C11—N2—C15	5.9 (3)
C2—C1—N1—C5	−179.53 (15)	C12—C11—N2—C15	−176.01 (16)
C10—C5—N1—C1	5.1 (3)	C20—C15—N2—C11	−1.6 (3)
C6—C5—N1—C1	−175.47 (15)	C16—C15—N2—C11	177.28 (16)

### Hydrogen-bond geometry (Å, °)

**Table:**

*D*—H···*A*	*D*—H	H···*A*	*D*···*A*	*D*—H···*A*
O2—H2A···O1	0.82	1.69	2.5080 (15)	175
O5—H5A···O4	0.82	1.72	2.5332 (17)	175
N1—H1N···O3^i^	0.77 (2)	2.30 (2)	3.0356 (18)	161.2 (19)
N2—H2N···O6^ii^	0.81 (2)	2.481 (19)	3.0775 (19)	131.3 (16)
N1—H1N···Cl1	0.77 (2)	2.56 (2)	2.9628 (14)	114.2 (17)
N2—H2N···Cl4	0.81 (2)	2.519 (18)	2.9476 (14)	114.3 (15)
C2—H2···O3^i^	0.93	2.29	3.123 (2)	149
C12—H12···O2	0.93	2.47	3.330 (2)	154

Symmetry codes: (i) −*x*+1, *y*+1/2, −*z*+3/2; (ii) −*x*, *y*+1/2, −*z*+3/2.

### Table 2 Table 2. Halogen-bond geometry (Å, °).

**Table:**

C–Cl···O	Cl···O	C–Cl···O
C9–Cl3···O6^iii^	3.1267 (15)	158.99 (6)
C16–Cl4···O1	3.0523 (12)	159.62 (7)

Symmetry code: (iii) x, -y+1/2, z-1/2.
